# 4,4′,6,6′-Tetra­methyl-2,2′-bipyrimidine hexa­hydrate

**DOI:** 10.1107/S1600536809010095

**Published:** 2009-03-25

**Authors:** Yanni Ma, Le Zhou, Dongsheng Deng, Baoming Ji

**Affiliations:** a Northwest Agriculture and Forest University, Yangling 712100, People’s Republic of China; b College of Chemistry and Chemical Engineering, Luoyang Normal University, Luoyang 471022, People’s Republic of China

## Abstract

In the title compound, C_12_H_14_N_4_·6H_2_O, the two pyrimidine rings make a dihedral angle of 5.285 (6)°. Inter­molecular O—H⋯O hydrogen bonds link the six water mol­ecules, generating edge-fused four-, five- or six-membered ring motifs and forming two-dimensional sheets. The sheets are stabilized by the formation of O—H⋯N hydrogen bonds between the water mol­ecules and the bipyrimidine mol­ecules, resulting in a three-dimensional network.

## Related literature

For 2,2′-bipyrimidine and its derivatives, see: Ji *et al.* (2000 ▶); Baumann *et al.* (1998 ▶). For hydrogen-bonded water clusters, see: Buck & Huisken (2000 ▶); Lakshminarayanan *et al.* (2006 ▶). For water–water inter­actions in bulk water or ice, see: Zhang *et al.* (2005 ▶). For bond lengths and angles, see: Berg *et al.* (2002 ▶). For the preparation of the compound by the Ullmann coupling method, see: Vlad & Horvath (2002 ▶).
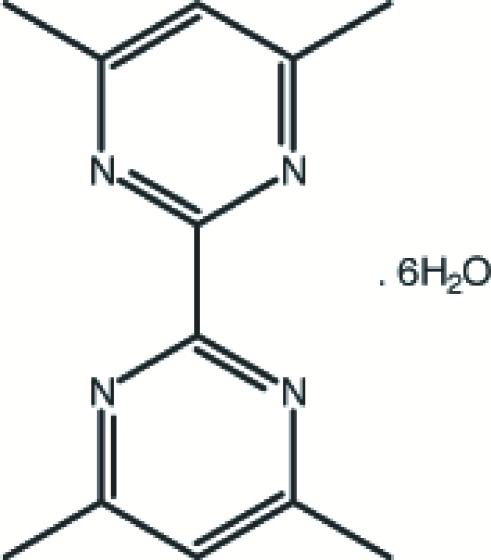


## Experimental

### 

#### Crystal data

C_12_H_14_N_4_·6H_2_O
*M* *_r_* = 322.37
Triclinic, 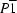

*a* = 6.8622 (19) Å
*b* = 11.098 (3) Å
*c* = 11.750 (3) Å
α = 98.233 (3)°
β = 91.774 (4)°
γ = 102.599 (4)°
*V* = 862.4 (4) Å^3^
*Z* = 2
Mo *K*α radiation
μ = 0.10 mm^−1^
*T* = 296 K
0.41 × 0.31 × 0.21 mm

#### Data collection

Bruker APEXII CCD area-detector diffractometer
Absorption correction: multi-scan (*SADABS*; Sheldrick, 1996 ▶) *T*_min_ = 0.961, *T*_max_ = 0.980
6492 measured reflections
3196 independent reflections
2026 reflections with *I* > 2σ(*I*)
*R*_int_ = 0.023

#### Refinement

*R*[*F* ^2^ > 2σ(*F* ^2^)] = 0.047
*wR*(*F* ^2^) = 0.148
*S* = 1.04
3196 reflections
204 parameters
H-atom parameters constrained
Δρ_max_ = 0.23 e Å^−3^
Δρ_min_ = −0.15 e Å^−3^

### 

Data collection: *APEX2* (Bruker, 2004 ▶); cell refinement: *SAINT* (Bruker, 2004 ▶); data reduction: *SAINT*; program(s) used to solve structure: *SHELXS97* (Sheldrick, 2008 ▶); program(s) used to refine structure: *SHELXS97* (Sheldrick, 2008 ▶); molecular graphics: *SHELXTL* (Sheldrick, 2008 ▶) and *DIAMOND* (Brandenburg, 2006 ▶); software used to prepare material for publication: *SHELXTL* and *PLATON* (Spek, 2009 ▶).

## Supplementary Material

Crystal structure: contains datablocks global, I. DOI: 10.1107/S1600536809010095/at2743sup1.cif

Structure factors: contains datablocks I. DOI: 10.1107/S1600536809010095/at2743Isup2.hkl

Additional supplementary materials:  crystallographic information; 3D view; checkCIF report

## Figures and Tables

**Table 1:** Hydrogen-bond geometry (Å, °)

*D*—H⋯*A*	*D*—H	H⋯*A*	*D*⋯*A*	*D*—H⋯*A*
O1—H1*W*⋯O3^i^	0.84	2.08	2.914 (2)	172
O1—H2*W*⋯O6	0.84	2.00	2.837 (2)	175
O2—H3*W*⋯N2	0.83	2.20	2.995 (2)	158
O2—H3*W*⋯N1	0.83	2.49	3.083 (2)	129
O2—H4*W*⋯O5^ii^	0.84	2.04	2.872 (2)	167
O3—H5*W*⋯O5^iii^	0.83	2.04	2.847 (2)	163
O3—H6*W*⋯O2	0.83	2.01	2.832 (2)	177
O4—H7*W*⋯O2	0.84	2.01	2.841 (2)	180
O4—H8*W*⋯O1^iv^	0.84	1.92	2.755 (2)	171
O5—H9*W*⋯N4^v^	0.83	2.31	3.007 (2)	142
O5—H9*W*⋯N3^v^	0.83	2.46	3.196 (2)	149
O5—H10*W*⋯O3	0.83	2.04	2.849 (2)	166
O6—H11*W*⋯O4^i^	0.83	2.05	2.851 (2)	162
O6—H12*W*⋯O4	0.84	2.06	2.886 (2)	173

Symmetry codes: (i) 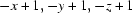; (ii) 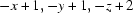; (iii) 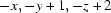; (iv) 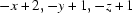; (v) 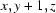.

**Fig. 1.**
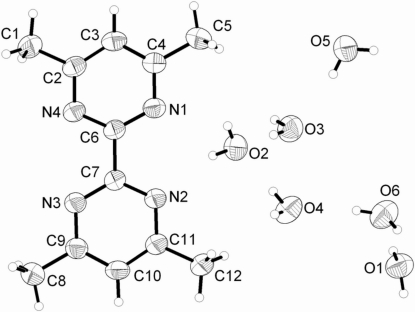
View of the title molecular structure with atom numbering scheme and 30% probability displacement ellipsoids for non-hydrogen atoms.

**Fig. 2.**
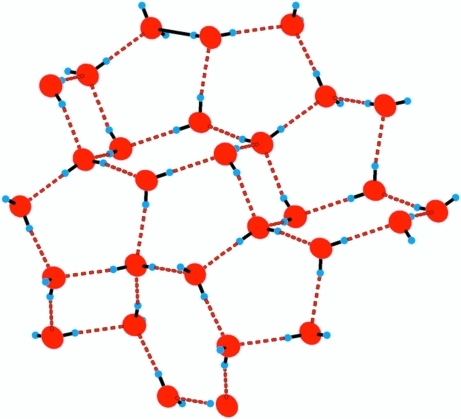
View of the two-dimensional sheet constructed by the lattice water molecules (O—H···O hydrogen bonds are represented as dashed lines).

## supplementary crystallographic information

### Comment

2,2'-Bipyrimidine and its derivatives have been used as the ligands in inorganic and organometallic chemistry (Ji *et al.* 2000; Baumann *et al.* 1998). On the other hand, the investigations of hydrogen-bonded water clusters in compound have recently attracted a great deal of interest (Buck *et al.* 2000; Lakshminarayanan *et al.* 2006). These studies can provided clues to understand the nature of water-water interactions in bulk water or ice (Zhang *et al.* 2005). In view of the importance of these compound, we herein report the synthesis and crystal structure of the title compound.

The molecule of the title compound (Fig. 1.), is built up form one pyrimidine ring connected to the other pyrimidine ring through the 2 and 2' carbon atoms, in which the bond lengths and angles are within ranges as reported by Berg *et al.* (2002). In the crystal structure, the four substituent methyl groups lie in the corresponding pyrimidine ring plane, respectively. And, the dihedral angle between the two pyrimidine rings is 5.285 (6)°. It must be pointed out that the striking feature of the title compound is the interesting arrangement of the six water molecules, which connected each other by the formation of intermolecular O—H···O hydrogen bonds, generating the edge-fused four-, five-, or six-membered ring motifs, to form a two-dimensional sheet (Fig.2.). Interestingly, every water O atom in the sheet is tri-coordination, which unlike the water at the surface of ice or in liquid water shows four coordination. Furthermore, the sheets are anchored in four nitrogen atoms of the titlelte molecule by the formation of O—H···N hydrogen bonds, resulting in a three-dimensional network, in which these hydrogen bonding interactions, with O—H···O hydrogen bonds may be effective in the stabilization of the crystal packing. Detail hydrogen bonds are given in Table 1.

### Experimental

The title compound was prepared according to the reported Ullmann coupling method (Vlad & Horvath, 2002). Under nitrogen-protected, 4,6-dimethyl-2-iodopyrimidine (351 mg, 1.5 mmol), absolute DMF (2.0 ml) and activated copper powder (508 mg, 8.0 mmol) were placed in a 25 ml flask. The reaction mixture was heated to 358 K with vigorous stirring. After 4 h, 127 mg (2 mmol) of activated copper powder was added to the mixture. After another 3.5 h, the temperature was increased to 398 K and the stirring was continued for 2 h. The suspension was then cooled to 273 K, carefully drowned into a solution of 1.4 g potassium cyanide in 6 ml of 25% aqueous solution of ammonia, and filtered. The solid residue on the filter was extracted with the same amount of cyanide solution and filtered again. The combined filtrates were treated with 58 mg of potassium cyanide and extracted with chloroform (5 times 20 ml). Washed with water and dried. Recrystallization of the crude product from ethyl acetate-petroleum ether gave 81 mg. The crystalline compound was luckily obtained by the reaction of the title compound with NdCl_3_ under the hydrothermal condition.

### Refinement

All H atoms were positioned geometrically and treated as riding, with C—H bond lengths constrained to 0.93 Å (aromatic CH), 0.96 Å (methyl CH_3_), and 0.83 or 0.84 Å (OH), and with U_ĩso_(H) = 1.2U_eq_(C) or 1.5U_eq_(methylene C or OH).

### Crystal data

**Table:**

C_12_H_14_N_4_·6H_2_O	*Z* = 2
*M**_r_* = 322.37	*F*(000) = 348
Triclinic, *P*1	*D*_x_ = 1.241 Mg m^−^^3^
Hall symbol: -P 1	Mo *K*α radiation, λ = 0.71073 Å
*a* = 6.8622 (19) Å	Cell parameters from 1270 reflections
*b* = 11.098 (3) Å	θ = 1.0–1.0°
*c* = 11.750 (3) Å	µ = 0.10 mm^−^^1^
α = 98.233 (3)°	*T* = 296 K
β = 91.774 (4)°	Block, colourless
γ = 102.599 (4)°	0.41 × 0.31 × 0.21 mm
*V* = 862.4 (4) Å^3^	

### Data collection

**Table:**

Bruker APEXII CCD area-detector diffractometer	3196 independent reflections
Radiation source: fine-focus sealed tube	2026 reflections with *I* > 2σ(*I*)
graphite	*R*_int_ = 0.023
φ and ω scans	θ_max_ = 25.5°, θ_min_ = 2.4°
Absorption correction: multi-scan (*SADABS*; Sheldrick, 1996)	*h* = −8→8
*T*_min_ = 0.961, *T*_max_ = 0.980	*k* = −13→13
6492 measured reflections	*l* = −14→14

### Refinement

**Table:**

Refinement on *F*^2^	Secondary atom site location: difference Fourier map
Least-squares matrix: full	Hydrogen site location: inferred from neighbouring sites
*R*[*F*^2^ > 2σ(*F*^2^)] = 0.047	H-atom parameters constrained
*wR*(*F*^2^) = 0.148	*w* = 1/[σ^2^(*F*_o_^2^) + (0.074*P*)^2^ + 0.0388*P*] where *P* = (*F*_o_^2^ + 2*F*_c_^2^)/3
*S* = 1.04	(Δ/σ)_max_ < 0.001
3196 reflections	Δρ_max_ = 0.23 e Å^−^^3^
204 parameters	Δρ_min_ = −0.15 e Å^−^^3^
0 restraints	Extinction correction: *SHELXS97* (Sheldrick, 2008), Fc^*^=kFc[1+0.001xFc^2^λ^3^/sin(2θ)]^-1/4^
Primary atom site location: structure-invariant direct methods	Extinction coefficient: 0.045 (6)

### Special details

**Table:**

Geometry. All e.s.d.'s (except the e.s.d. in the dihedral angle between two l.s. planes)are estimated using the full covariance matrix. The cell e.s.d.'s are takeninto account individually in the estimation of e.s.d.'s in distances, anglesand torsion angles; correlations between e.s.d.'s in cell parameters are onlyused when they are defined by crystal symmetry. An approximate (isotropic)treatment of cell e.s.d.'s is used for estimating e.s.d.'s involving l.s. planes.
Refinement. Refinement of *F*^2^ against ALL reflections. The weighted *R*-factor *wR* andgoodness of fit *S* are based on *F*^2^, conventional *R*-factors *R* are basedon *F*, with *F* set to zero for negative *F*^2^. The threshold expression of*F*^2^ > σ(*F*^2^) is used only for calculating *R*-factors(gt) *etc*. and isnot relevant to the choice of reflections for refinement. *R*-factors basedon *F*^2^ are statistically about twice as large as those based on *F*, and *R*-factors based on ALL data will be even larger.

### Fractional atomic coordinates and isotropic or equivalent isotropic displacement parameters (Å^2^)

**Table:**

	*x*	*y*	*z*	*U*_iso_*/*U*_eq_	
O1	1.0078 (2)	0.66049 (15)	0.38126 (13)	0.0642 (5)	
H1W	0.9576	0.6341	0.3142	0.096*	
H2W	0.9194	0.6501	0.4285	0.096*	
O2	0.4638 (2)	0.32153 (14)	0.82784 (13)	0.0576 (5)	
H3W	0.3978	0.2491	0.8284	0.086*	
H4W	0.5591	0.3379	0.8787	0.086*	
O3	0.1279 (3)	0.42962 (14)	0.85957 (13)	0.0620 (5)	
H5W	0.0432	0.3909	0.8985	0.093*	
H6W	0.2226	0.3952	0.8493	0.093*	
O4	0.6255 (3)	0.40256 (16)	0.62491 (14)	0.0672 (5)	
H7W	0.5777	0.3790	0.6846	0.101*	
H8W	0.7336	0.3788	0.6155	0.101*	
O5	0.2031 (2)	0.66114 (14)	1.01180 (14)	0.0624 (5)	
H9W	0.2119	0.7281	0.9869	0.094*	
H10W	0.1897	0.6013	0.9586	0.094*	
O6	0.7043 (3)	0.63916 (16)	0.54052 (14)	0.0705 (5)	
H11W	0.6024	0.6411	0.5015	0.106*	
H12W	0.6844	0.5744	0.5710	0.106*	
N1	0.2840 (3)	0.14305 (16)	0.99505 (14)	0.0417 (4)	
N2	0.2855 (3)	0.04691 (15)	0.77127 (13)	0.0417 (4)	
N3	0.2336 (2)	−0.15832 (15)	0.82104 (14)	0.0403 (4)	
N4	0.2327 (2)	−0.06158 (15)	1.04606 (14)	0.0407 (4)	
C1	0.1870 (4)	−0.1038 (2)	1.24073 (18)	0.0559 (6)	
H1A	0.0530	−0.1535	1.2264	0.084*	
H1B	0.2033	−0.0587	1.3178	0.084*	
H1C	0.2801	−0.1572	1.2319	0.084*	
C2	0.2256 (3)	−0.0134 (2)	1.15686 (17)	0.0414 (5)	
C3	0.2480 (3)	0.1139 (2)	1.18979 (17)	0.0452 (5)	
H3	0.2434	0.1471	1.2667	0.054*	
C4	0.2775 (3)	0.19090 (19)	1.10592 (17)	0.0432 (5)	
C5	0.3011 (4)	0.3293 (2)	1.1331 (2)	0.0632 (7)	
H5A	0.4190	0.3704	1.1002	0.095*	
H5B	0.3133	0.3543	1.2152	0.095*	
H5C	0.1861	0.3522	1.1015	0.095*	
C6	0.2612 (3)	0.01975 (18)	0.97125 (16)	0.0365 (5)	
C7	0.2621 (3)	−0.03399 (18)	0.84670 (16)	0.0363 (5)	
C8	0.1946 (4)	−0.3443 (2)	0.6791 (2)	0.0617 (7)	
H8A	0.3139	−0.3691	0.7017	0.093*	
H8B	0.1661	−0.3685	0.5975	0.093*	
H8C	0.0846	−0.3845	0.7189	0.093*	
C9	0.2245 (3)	−0.20604 (19)	0.70895 (17)	0.0432 (5)	
C10	0.2398 (3)	−0.1290 (2)	0.62551 (18)	0.0496 (6)	
H10	0.2286	−0.1625	0.5477	0.060*	
C11	0.2719 (3)	−0.00161 (19)	0.65939 (17)	0.0449 (5)	
C12	0.2935 (4)	0.0891 (2)	0.57525 (19)	0.0644 (7)	
H12A	0.1977	0.1402	0.5887	0.097*	
H12B	0.2705	0.0442	0.4982	0.097*	
H12C	0.4262	0.1413	0.5849	0.097*	

### Atomic displacement parameters (Å^2^)

**Table:**

	*U*^11^	*U*^22^	*U*^33^	*U*^12^	*U*^13^	*U*^23^
O1	0.0689 (12)	0.0708 (11)	0.0513 (10)	0.0119 (9)	0.0050 (8)	0.0098 (8)
O2	0.0637 (11)	0.0499 (9)	0.0589 (10)	0.0082 (8)	0.0062 (8)	0.0138 (8)
O3	0.0686 (11)	0.0632 (11)	0.0583 (10)	0.0187 (8)	0.0111 (8)	0.0151 (8)
O4	0.0691 (12)	0.0825 (12)	0.0556 (10)	0.0191 (9)	0.0074 (8)	0.0251 (9)
O5	0.0743 (12)	0.0495 (9)	0.0617 (10)	0.0089 (8)	0.0045 (9)	0.0101 (8)
O6	0.0698 (12)	0.0794 (12)	0.0626 (11)	0.0096 (9)	0.0030 (9)	0.0231 (9)
N1	0.0440 (10)	0.0440 (10)	0.0355 (9)	0.0073 (8)	0.0036 (8)	0.0043 (8)
N2	0.0505 (11)	0.0413 (10)	0.0332 (9)	0.0100 (8)	0.0055 (8)	0.0052 (8)
N3	0.0430 (10)	0.0412 (10)	0.0365 (9)	0.0090 (8)	0.0047 (8)	0.0057 (8)
N4	0.0407 (10)	0.0472 (10)	0.0349 (9)	0.0105 (8)	0.0049 (7)	0.0076 (8)
C1	0.0706 (16)	0.0596 (15)	0.0415 (13)	0.0177 (12)	0.0136 (11)	0.0141 (11)
C2	0.0357 (11)	0.0537 (13)	0.0351 (11)	0.0096 (10)	0.0038 (9)	0.0082 (9)
C3	0.0448 (13)	0.0571 (14)	0.0328 (11)	0.0124 (10)	0.0058 (9)	0.0023 (10)
C4	0.0422 (12)	0.0469 (12)	0.0387 (12)	0.0091 (10)	0.0017 (9)	0.0021 (9)
C5	0.0879 (19)	0.0528 (15)	0.0461 (14)	0.0161 (13)	0.0047 (13)	−0.0024 (11)
C6	0.0326 (11)	0.0438 (12)	0.0330 (11)	0.0083 (9)	0.0031 (8)	0.0060 (9)
C7	0.0317 (10)	0.0417 (11)	0.0356 (11)	0.0079 (9)	0.0035 (8)	0.0067 (9)
C8	0.0899 (19)	0.0485 (14)	0.0475 (14)	0.0197 (13)	0.0058 (13)	0.0033 (11)
C9	0.0462 (13)	0.0427 (12)	0.0397 (12)	0.0088 (9)	0.0047 (9)	0.0044 (9)
C10	0.0640 (15)	0.0482 (13)	0.0322 (11)	0.0072 (11)	0.0025 (10)	0.0002 (10)
C11	0.0523 (13)	0.0462 (13)	0.0361 (11)	0.0098 (10)	0.0050 (10)	0.0078 (9)
C12	0.103 (2)	0.0525 (15)	0.0378 (13)	0.0139 (14)	0.0069 (13)	0.0121 (11)

### Geometric parameters (Å, °)

**Table:**

O1—H1W	0.8358	C1—H1B	0.9600
O1—H2W	0.8355	C1—H1C	0.9600
O2—H3W	0.8334	C2—C3	1.383 (3)
O2—H4W	0.8431	C3—C4	1.386 (3)
O3—H5W	0.8342	C3—H3	0.9300
O3—H6W	0.8269	C4—C5	1.496 (3)
O4—H7W	0.8351	C5—H5A	0.9600
O4—H8W	0.8443	C5—H5B	0.9600
O5—H9W	0.8278	C5—H5C	0.9600
O5—H10W	0.8314	C6—C7	1.500 (3)
O6—H11W	0.8302	C8—C9	1.492 (3)
O6—H12W	0.8353	C8—H8A	0.9600
N1—C6	1.330 (2)	C8—H8B	0.9600
N1—C4	1.340 (3)	C8—H8C	0.9600
N2—C7	1.338 (2)	C9—C10	1.383 (3)
N2—C11	1.340 (3)	C10—C11	1.379 (3)
N3—C7	1.339 (2)	C10—H10	0.9300
N3—C9	1.341 (3)	C11—C12	1.498 (3)
N4—C6	1.337 (2)	C12—H12A	0.9600
N4—C2	1.341 (3)	C12—H12B	0.9600
C1—C2	1.493 (3)	C12—H12C	0.9600
C1—H1A	0.9600		
			
H1W—O1—H2W	110.2	H5A—C5—H5C	109.5
H3W—O2—H4W	109.0	H5B—C5—H5C	109.5
H5W—O3—H6W	111.2	N1—C6—N4	126.97 (18)
H7W—O4—H8W	108.4	N1—C6—C7	116.44 (17)
H9W—O5—H10W	111.6	N4—C6—C7	116.57 (18)
H11W—O6—H12W	109.9	N3—C7—N2	126.13 (18)
C6—N1—C4	116.54 (17)	N3—C7—C6	117.13 (17)
C7—N2—C11	116.80 (17)	N2—C7—C6	116.70 (17)
C7—N3—C9	116.70 (17)	C9—C8—H8A	109.5
C6—N4—C2	116.31 (18)	C9—C8—H8B	109.5
C2—C1—H1A	109.5	H8A—C8—H8B	109.5
C2—C1—H1B	109.5	C9—C8—H8C	109.5
H1A—C1—H1B	109.5	H8A—C8—H8C	109.5
C2—C1—H1C	109.5	H8B—C8—H8C	109.5
H1A—C1—H1C	109.5	N3—C9—C10	120.62 (19)
H1B—C1—H1C	109.5	N3—C9—C8	117.30 (18)
N4—C2—C3	120.80 (18)	C10—C9—C8	122.07 (19)
N4—C2—C1	116.81 (19)	C11—C10—C9	118.96 (19)
C3—C2—C1	122.37 (19)	C11—C10—H10	120.5
C2—C3—C4	118.69 (19)	C9—C10—H10	120.5
C2—C3—H3	120.7	N2—C11—C10	120.71 (18)
C4—C3—H3	120.7	N2—C11—C12	116.59 (19)
N1—C4—C3	120.68 (19)	C10—C11—C12	122.71 (19)
N1—C4—C5	116.81 (19)	C11—C12—H12A	109.5
C3—C4—C5	122.50 (19)	C11—C12—H12B	109.5
C4—C5—H5A	109.5	H12A—C12—H12B	109.5
C4—C5—H5B	109.5	C11—C12—H12C	109.5
H5A—C5—H5B	109.5	H12A—C12—H12C	109.5
C4—C5—H5C	109.5	H12B—C12—H12C	109.5
			
C6—N4—C2—C3	−0.3 (3)	C11—N2—C7—N3	2.5 (3)
C6—N4—C2—C1	178.04 (18)	C11—N2—C7—C6	−175.32 (18)
N4—C2—C3—C4	0.2 (3)	N1—C6—C7—N3	−178.13 (16)
C1—C2—C3—C4	−178.1 (2)	N4—C6—C7—N3	0.3 (3)
C6—N1—C4—C3	−0.1 (3)	N1—C6—C7—N2	−0.1 (3)
C6—N1—C4—C5	−179.45 (19)	N4—C6—C7—N2	178.33 (16)
C2—C3—C4—N1	0.1 (3)	C7—N3—C9—C10	−1.4 (3)
C2—C3—C4—C5	179.3 (2)	C7—N3—C9—C8	179.46 (19)
C4—N1—C6—N4	0.0 (3)	N3—C9—C10—C11	2.3 (3)
C4—N1—C6—C7	178.16 (17)	C8—C9—C10—C11	−178.5 (2)
C2—N4—C6—N1	0.3 (3)	C7—N2—C11—C10	−1.4 (3)
C2—N4—C6—C7	−177.94 (17)	C7—N2—C11—C12	178.63 (19)
C9—N3—C7—N2	−1.1 (3)	C9—C10—C11—N2	−0.9 (3)
C9—N3—C7—C6	176.71 (17)	C9—C10—C11—C12	179.1 (2)

### Hydrogen-bond geometry (Å, °)

**Table:**

*D*—H···*A*	*D*—H	H···*A*	*D*···*A*	*D*—H···*A*
O1—H1W···O3^i^	0.84	2.08	2.914 (2)	172
O1—H2W···O6	0.84	2.00	2.837 (2)	175
O2—H3W···N2	0.83	2.20	2.995 (2)	158
O2—H3W···N1	0.83	2.49	3.083 (2)	129
O2—H4W···O5^ii^	0.84	2.04	2.872 (2)	167
O3—H5W···O5^iii^	0.83	2.04	2.847 (2)	163
O3—H6W···O2	0.83	2.01	2.832 (2)	177
O4—H7W···O2	0.84	2.01	2.841 (2)	180
O4—H8W···O1^iv^	0.84	1.92	2.755 (2)	171
O5—H9W···N4^v^	0.83	2.31	3.007 (2)	142
O5—H9W···N3^v^	0.83	2.46	3.196 (2)	149
O5—H10W···O3	0.83	2.04	2.849 (2)	166
O6—H11W···O4^i^	0.83	2.05	2.851 (2)	162
O6—H12W···O4	0.84	2.06	2.886 (2)	173

Symmetry codes: (i) −*x*+1, −*y*+1, −*z*+1; (ii) −*x*+1, −*y*+1, −*z*+2; (iii) −*x*, −*y*+1, −*z*+2; (iv) −*x*+2, −*y*+1, −*z*+1; (v) *x*, *y*+1, *z*.
